# [Retracted] Effects and mechanism of Xin Mai Jia in a rabbit model of atherosclerosis

**DOI:** 10.3892/etm.2025.12869

**Published:** 2025-04-14

**Authors:** Fan-Rong Zhao, Jun-Xiu Lu, Mei Jia, Ya-Ling Yin, Heng-Tian Qi, Mo-Li Zhu, Li-Juan Ma, Le-Le Qiu, Guang-Ming Wan, Guang-Rui Wan

Exp Ther Med 10:1627–1634, 2015; DOI: 10.3892/etm.2015.2774

Following the publication of this article, a concerned reader drew to the Editor’s attention that, regarding the vascular images presented in Figs. 3, 4 and 6, certain of the images selected within these figures were either overlapping or duplicated, where the results from differently performed experiments were intended to have been shown. Moreover, two rows of data in Table II, reporting the data for different (i.e., the NC and MXG) groups, were entirely matching with respect to the whole blood viscosity measurements, which was a rather improbable outcome.

After having conducted an internal investigation, the Editor of *Experimental and Therapeutic Medicine* has reached the conclusion that Figs. 3, 4 and 6 and Table II contained an unacceptable number of errors with respect to the compilation of the images/data. Therefore, on the grounds of a lack of confidence in the integrity of these data, the Editor has decided that the article should be retracted from the publication. The authors were asked for an explanation to account for these concerns, but the Editorial Office did not receive a reply. The Editor apologizes to the readership for any inconvenience caused, and thanks the interested reader for drawing this matter to our attention.

